# In the mind of the beholder: The effects of familiarisation on the perception of atypical infant facial configurations

**DOI:** 10.1371/journal.pone.0289057

**Published:** 2023-07-25

**Authors:** Benjamin W. Hunt, Holly Rayson, Colin Bannard, Leonardo De Pascalis

**Affiliations:** 1 Department of Psychology, University of Liverpool, Liverpool, United Kingdom; 2 Institut des Sciences Cognitives Marc Jeannerod, Centre National de la Recherche Scientifique, Université Claude Bernard, Lyon 1, France; 3 Department of Linguistics and English Language, University of Manchester, Manchester, United Kingdom; 4 Department of Psychology, University of Bologna, Bologna, Italy; Northumbria University, UNITED KINGDOM

## Abstract

Infant facial attractiveness is an important facilitator for adult-infant caregiving behaviour. Disruption to typical infant facial configurations can, however, attenuate their perceived attractiveness, as rated by adult observers. Previous research has either focused on how ratings are affected by observer characteristics (e.g., male/female), or alterations to infant faces, either experimentally, or naturalistically induced, such as the presence of a cleft lip. Little research has however been conducted on the effects of observer experience on adult ratings of infant facial attractiveness. Such effects could inform clinical work and policies aimed at promoting positive perception of facial malformations. The present study thus explored the effects of familiarisation on how typical and atypical infant facial configurations are evaluated by adults. We recruited two groups of female participants and compared their subjective attractiveness ratings of infant faces (24 typical and 24 cleft-affected), at baseline, and at one-week post-test. Between the two assessments, one group (*n* = 41) underwent a week-long training phase, where they were familiarised with cleft lip/palate-related visual and informational stimuli, while the control group (*n* = 44) received no training. Significantly higher ratings were provided for faces of typically developing versus cleft-affected infants by both groups of participants at baseline. At post-test, this pattern of ratings was repeated in participants belonging to the control group, while familiarised participants showed an increase, compared to baseline, in their ratings of cleft-affected faces and no difference between their evaluation of the latter and that of typically developing faces. These findings extend our understanding of the observer’s experience in the evaluation of infant faces, beyond the effects of the structural characteristics of the observed faces. Results also highlight familiarity as a potentially protective influence against the negative consequences of alterations to typical facial configurations, suggesting avenues for intervention in supporting adult caregivers in the context of neonatal facial malformations.

## Introduction

The ability of caregivers to respond sensitively to infant communicative cues has been shown to be a powerful promoter of infant development and optimal infant outcomes [1–4]. A prerequisite of this ability is represented by caregivers being able to, first of all, perceive these infant cues, having focused their attention toward them. Research has found that adults show a preference for infant versus adult faces [5, 6], an effect that is likely to be related to facilitating caregiving in human dyads [7]. Among infants, faces may also vary in their perceived degree of “cuteness”, which may further influence observers’ reactions. Cuter infants are looked at for longer [8], and are even perceived as being more capable (e.g., having increased communicative and social skills and cognitive abilities) than less cute infants [9]. Of note, adult perceptions of infant cuteness have been found to be affected by infant age and gender, with younger age [10–12] and female gender [10, 12] being associated with higher cuteness scores. Interestingly, previous research using static image face stimuli has also found adult observers to be sensitive to subtle cues, such as variations to gaze direction, with preferences being displayed for images when the target’s eyes are closer to directly engaging the participants’ gaze, and images showing averted gaze being rated as less attractive [13, 14]. These latter findings emerge from studies employing images of adult faces, but they may plausibly apply to responses to infant faces, and they speak to the numerous factors affecting the perception of facial attractiveness.

Kindchenschema, also referred to as “baby schema”, is a term coined by the ethologist Konrad Lorenz [15], to refer to “babyish” features such as large eyes and cheeks, a small nose, and a high forehead, with infants who are considered higher on this baby schema spectrum also being the ones who are considered to be “cuter” [16]. These features are thought to signal increased vulnerability [17], evoking parental caregiving responses, thereby increasing the infant’s chances of survival. Adult processing of infant faces would thus appear to play a particularly important role in inducing caretaking behaviour [18], and any alteration to the infant’s facial configuration is likely to result in disrupted visual processing of the infant face [7, 19], and a subsequent change in how infant appearance is perceived.

Adult reactions to variation in baby schema may be assessed via manipulation of infant features, for example, by increasing the proportionate size of the eyes in relation to the head and measuring participant response [20]. Studies using this methodology typically report higher attractiveness ratings for images of infants higher in baby schema features [16, 21], with a greater activation of the nucleus accumbens region of the brain—a structure associated with reward and appetitive motivation—also found in response to images of infants with exaggerated baby schema facial features compared to attenuated and unmanipulated baby schema features [22].

Another body of research on infant face processing focuses on naturally occurring alterations to the baby schema, which provide the scientific community with the opportunity to investigate disruptions to visual processes in a naturalistic setting. An often-studied example of such disruptions is represented by cleft lip and/or palate (CLP), the most common congenital facial anomaly, with an incidence rate of approximately 1 in 700 births in the UK [23]. Indeed, with regard to neurophysiological perception of atypical infant faces, Parsons et al. [24] found significantly reduced activity in the right fusiform face area, an aspect of the neural visual system putatively involved in processing faces, when participants were presented with images of infants affected by a cleft, compared to images of typically developing (TD) infants. Additionally, research using electroencephalography found reduced P1 and N170 amplitude (event related potential components associated with processing of faces [25]), in response to faces of infants affected by clefts compared to TD infants, suggesting the presence of a facial abnormality disrupts “normative” processing of infant faces [26]. Studies using subjective ratings as an outcome have found that, compared to TD infants, CLP infants receive lower “cuteness,” attractiveness, and caregiving ratings in hypothetical care provision scenarios [27, 28], are viewed for shorter durations [21, 29], and are rated as less desirable to look at [26]. The wealth of extant research on visual processing of infant faces with a cleft lip is likely a reflection of its relatively high incidence rate and naturalistic value in investigating infant face processing.

In addition to variations in the facial configuration and the “cuteness” of a target infant, the status of the individual perceiving the infant can also affect their reactions. Gender differences in the response to typical infant faces present themselves early in life. Female children (aged 11 to 14) tend to show a preference for babyish faces earlier than male children, who do not show this tendency for approximately another two years [30]. This effect remains until later in the lifespan, as the response to infant schema is more pronounced in women than in men: females provide higher cuteness and caregiving ratings [16], and respond with more positive facial expressions [31] than do males. Additionally, age of the perceiver and its relationship with the perception of infant faces has been explored in adults, with younger females perceiving infants as more attractive and being more sensitive to differences in infant “cuteness” than older females (e.g., [20, 32]).

While the studies mentioned above explored the influence of pre-existing and established observer characteristics on how infant faces are perceived and appraised, very few studies (see [33] for a notable exception) have investigated the effects of manipulating the perceiver’s experience, as related to the infant face being evaluated. This gap in knowledge is of particular potential importance, given recent contrasting findings in the literature, showing differing reactions in viewers with different levels of experience with the specific characteristics of the face being perceived. Specifically, when presented with images of cleft affected infants, naïve observers tend to fixate their gaze on the mouth area of the infant (e.g., [28], who also showed this to be more pronounced with more severe clefts, which also lead to lower cuteness ratings), and the time they spent visually attending to this area was found to negatively correlate with how attractive they found the cleft-affected face to be [28]. In a separate study, mothers of cleft affected infants were instead found to show a striking tendency to visually attend to facial areas other than the mouth area of their own infant, from the first weeks of the latter’s life [34], especially in the case of particularly severe clefts [4]. It would be reasonable to see this striking difference as tied to the several factors that differentiate a naive observer of a still picture of an infant face with a cleft lip from a mother interacting in real time with her infant born with the same kind of facial malformation. Aside from the notable difference in stimulus presentation, the latter group of individuals possesses several ‘nested’ characteristics known to affect the processing of infant related stimuli: thus, they are mothers of those specific infants they are looking at [35–38], mothers of infants born with a cleft lip [39], and mothers in general [40–42]. While recognising these group differences, and their probable role in the aforementioned difference in findings on naive observers *vs*. mothers, the present paper aims to focus on the "lowest common denominator" characteristic shared by the two groups, the simplest possible common aspect of their viewing experience, that of being adult observers gazing at an infant face with a cleft lip. One factor that sits within this common characteristic, but which differs in degree between the two groups, thus potentially contributing to their different processing of cleft-affected infant faces, is that of perceiver familiarity with the specific nature of the stimulus.

In relation to the aforementioned difference in gaze behaviour between naïve observers and mothers, when viewing faces of infants with CLP, the role of familiarity may take two routes. On the one hand, mothers are, generally, frequently exposed to their infant’s face, its characteristics, and the details of the CLP, where this is present; on the other hand, an idiosyncrasy of the CLP diagnostic process may be, in part, responsible for maternal familiarisation with CLP related stimuli. Seventy-eight percent of CLP diagnoses are made during the 22-week prenatal scan [43]. Upon this diagnosis, additional preparations are made for the expectant woman prenatally, such as being placed in contact with members of a specialist cleft team [44]. Official materials provided by healthcare providers, voluntary organisations (see the Cleft Lip and Palate Association (CLAPA); for a UK-based example), and the existence of social media groups (e.g., parents and carers support groups; see also CLAPA) suggests that some degree of maternal familiarisation with CLP-related visual stimuli and factual information is likely taking place even pre-birth for a relatively large number of women whose to-be-born infant has received a CLP diagnosis, as they are being provided with materials, support, and advice that is likely to have a familiarising side effect, by introducing them to the characteristics of CLP.

Given the presence of these experiences in mothers, and their specific reactions to CLP exposure [34], the present study was primarily interested in exploring the effects of familiarisation on how typical and atypical infant face configurations are perceived and evaluated by female participants. To this end, a set of visual stimuli depicting infant faces with or without CLP was shown to two groups of female individuals with no family/personal history of CLP or familiarity with it, at two time points: in only one of the two groups, a week-long active familiarisation (AF) training module was provided between the two assessments, based on the kind of visual and informational material mothers of infants with CLP receive prenatally.

Our research hypotheses were twofold: firstly, in line with previous research, it was predicted that images of TD infants would be rated significantly higher on indices of subjective attractiveness (“cuteness”) than the images of infants with CLP, at baseline. Secondly, it was predicted that, only for AF participants’ ratings of images containing CLP, “cuteness” ratings would be higher post-test than at baseline, due to the familiarising effect of the AF training. No difference between time points was expected for participants not receiving the AF training.

## Method

### Participants

One hundred and five adult females aged 18–28 (M = 19.36 ±1.39) were recruited, via opportunity sampling, from the University of Liverpool undergraduate and postgraduate student population (with the exception of two non-student participants, recruited via word of mouth). To be included, participants were required to be females, not to be parents, to have normal or corrected to normal vision, and to have no prior familiarity with infants born with CLP. Participants were randomly allocated using a random number generator into either the experimental group, who received the active familiarisation training, or the control group, who received no training. Although 105 participants were recruited, three participants dropped out of the study before its start, and 17 participants (8 from the control group, 9 from the experimental group) only completed the first of the two planned assessments. The data available from the 17 participants who only provided baseline ratings were included in the analyses (although consistency of results with the exclusion of these participants was checked and reported below). Eighty-five participants (experimental group, *n* = 41; control group, *n* = 44) completed both the baseline and post-test assessments.

Sample size analysis for the current study was conducted using the GLIMMPSE 3.0 Sample Size Software [45]. Specifying the design for the present study, a statistical power of .95, and a significance value of .05, the total suggested sample size was 76 participants, for an effect size of f = .20. The research was conducted in accordance with the ethical standards outlined by the Helsinki Declaration of 1975, as revised in 2001 (World Medical Association, [46]). Ethical approval was received through the University of Liverpool Central Research Ethics Committee (CUREA; approval number: 7551) and written, informed consent was obtained from each participant prior to testing.

## Materials

### Infant face stimuli

**Fig 1:**
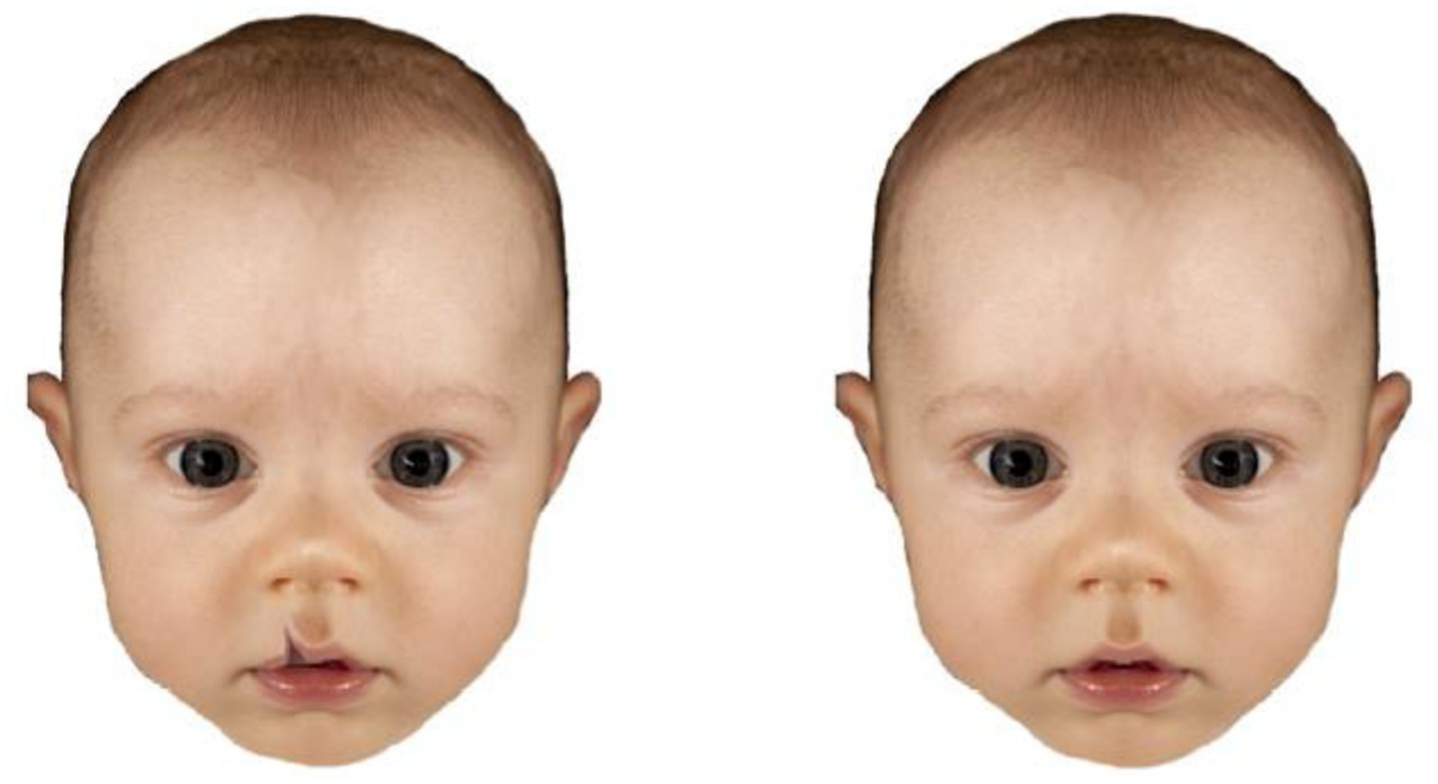
Example images of infants with, and without, cleft lip. Images taken with permission from Lewis et al. [27], adapted from [48].

Forty-eight images of typical infants ranging in age from 3–13 months (M = 7.79; sd = 3.28) were used, which were derived from Hildebrandt and Fitzgerald [10], who obtained parental consent to use the images for research purposes and provided information on the infant’s ages. These same 48 images were digitally modified by Lewis et al. [27], in order to make the infants appear to be affected by CLP. Example images of cleft lip were sourced online for their size and colour and blended with the original image so as to appear natural (see Fig 1). Approximately half of the mouths were flipped horizontally to ensure the horizontal location of the cleft was counterbalanced between faces. Images were standardised by removing backgrounds and adjusting in order to be equal in size. All images featured an infant with a neutral expression where the face could be seen clearly (i.e., not obscured by hair). The images were presented in Qualtrics [47].

### “Cuteness” ratings

Participants provided “cuteness” ratings for each infant face. The assessment was adapted from a study conducted by Lewis et al. [27], who assessed participants on indices of “cuteness” and “attractiveness.” A Likert scale was presented following each infant image, and participants selected a value ranging 1–7 in response to the question “How cute do you rate this infant?” (“Not cute at all” to “Extremely cute”) using a mouse click on a sliding scale. The question was presented on the same screen with the sliding scale situated immediately beneath it. Upon completion of the response, the screen automatically advanced to the next infant image. As for the images, the questions were also presented in Qualtrics [47], and appeared immediately after the relevant infant image, for each of the 48 trials.

### Cleft severity

Given the effect of cleft severity on infant cuteness perception, the 48 images of cleft affected infants were rated on a scale of severity (mild, moderate, and severe) by two independent raters who were blind to the aims of the study. Differences of opinion were resolved through discussion. Out of the three possible severity categories, seven images were rated as mild, 31 as moderate, and ten as severe.

### Gaze direction of infant images

To account for their possible variability and effect on participant ratings, estimates of infant gaze and head direction were derived using the OpenFace Facial Behaviour Analysis Toolkit [49]. This toolkit provided eye gaze direction in radians, on both horizontal and vertical planes. Head direction estimates provided head rotation in radians around three possible planar axes, indicating head pitch, yaw, and roll. To minimise the number of potential predictors in the model, estimates of general gaze and head off-centeredness were derived through summation of the absolute values of OpenFace estimates for gaze direction, and for head direction, respectively, with zero representing perfect centeredness.

### Active familiarisation (AF) training development

The AF training materials were created using informational and audio-visual material on infant CLP, using content that was more condensed but similar in nature to what is sought and received by pregnant mothers whose unborn infant is diagnosed with CLP. These materials were created using informational literature that is provided to mothers upon diagnosis of the cleft (either at 22-week scan or upon delivery of infant) such as educational leaflets created by Northwest England, The Isle of Man, and North Wales Cleft lip and Palate Network [50], and public health information websites such as the NHS (UK National Health System; [23]). Images and text information was also taken, with permission, from CLAPA, a charity with an online presence for families affected by infant CLP. The purpose of this audio-visual information, when in the public domain, is to provide information, advice, and support to those close to infants diagnosed with CLP.

Participants were required to access seven daily instalments, comprising approximately 20 slides per session, that were designed to influence the participants’ attitudes by increasing their awareness of the condition and its physical and medical characteristics through explicit exposure to a heavy load of visual stimulation and factual information. A typical slide comprised text of around 50 words, accompanied by an image of an infant born with CLP that took up approximately half the size of the entire slide. Most images were of the infant pre-surgery, however, where information was given about infants post-surgery, a small number (<11%) were images of children post-surgical procedure. Although the images used in the AF training showed infants of ages similar to the ones depicted in the images used in the rating task, these infants were not the same: AF participants did not view the images used in the rating task until subjected to said task. Of the total seven instalments, five were created by grouping similar information into discrete life events and/or characteristics related to experience of CLP (such as treatment and feeding). The remaining two instalments were adapted from personal stories that mothers of infants born with CLP had written and uploaded to the CLAPA website, in the form of support for other families of infants with CLP. A description of the components of the training can be found below. Materials were developed and presented using the Gorilla Experiment Builder [51].

#### Active familiarisation (AF) training components

1. *Diagnosis & Statistics*.
  Basic information about the appearance of clefts, the incidence rate of CLP in the UK, and the process by which the cleft is typically diagnosed (22-week ultrasound scan). At this point in the training, participants were provided with factual information about the causes of CLP; specifically, that they are largely unknown and unlikely to be related to the parents’ behaviour.
2. *Causes & Difficulties*.
  This instalment reiterated that causes of CLP are currently unknown, then provided details about some of the common difficulties that infants born with CLP experience during their development, such as difficulties with feeding, speech problems, and health issues such as glue ear (fluid entering the middle part of the ear canal) and dental problems. Information about dedicated cleft teams is also presented in this instalment, for example, that the family will be referred to specialist cleft surgeons, nurses, and restorative dentists.
3. *Treatment & Surgery*.
  As surgery is the principal form of treatment for CLP [52], details are given about the surgical procedure, the number of procedures the child may need to undergo, and the child’s appearance post-operation. In addition, information about follow-up treatment, such as orthodontic care and speech therapy is provided.
4. *Personal Story 1*.
  A patient story written by the mother of an infant born with undiagnosed CLP. The mother describes the shock of receiving the diagnosis immediately after the birth, and her disappointment when being told she would be unable to breastfeed her baby. The story was published on the CLAPA website.
5. *Feeding*.
  As there can be complications surrounding feeding when the infant is affected by CLP, this instalment informs the participants about the different solutions to said complications, such as expressing breast milk then bottle feeding with a specially modified teat. Other problems such as milk leaking from the infant’s nose while feeding, and their requirement to be “winded” more regularly are also explained.
6. *Future & Outlook*.
  This instalment provides information about how a child born with CLP might be affected in the future. Previously provided information about the requirement for speech therapy is briefly touched on, before moving onto topics such as attending school, dealing with peers, and responding to teasing.
7. *Personal Story 2*.
  A second patient story written by the mother of an infant affected by CLP. The mother received the diagnosis at the 22-week scan and describes her emotions (fear, guilt, anger) upon learning the news. She also describes how, contrary to professional advice, she searched for images and information about the condition using an internet search engine. The story was also published on the CLAPA website.

### Procedure

Participants were recruited from the University of Liverpool student body, through a university-based system promoting research participation (with the exception of two non-student participants, recruited via word of mouth). Once individuals had indicated their intention to participate, they were randomised to either the control or AF training group, and each received an email invitation that included a link to the study and a unique password that enabled access to the "cuteness" assessment. Having accessed the link, participants first received information about the study and provided signed informed consent, and then began the first stage of the study, namely the “cuteness” rating, delivered online via Qualtrics. Participants were exposed to 48 trials (24 TD images, 24 CLP images), with one trial consisting of viewing a single image for 10 seconds and providing a subjective “cuteness” rating of said image, as per Lewis et al. [27]. Images were counterbalanced so that none was seen twice (for instance, no participant viewed an image of the same infant both with and without a cleft or of the same infant twice). To ensure this, participants were presented at random with one of two versions of the task, each created with 48 images. Cleft affected infants in version A of the task appeared in version B as typical infants, and vice versa.

The “cuteness” question was presented after each image, with participants having no time limit to give their answers. Upon completion of the assessment, participants were provided with a debriefing document and reminded that they would receive an invitation to complete the same assessment in seven days’ time.

Following completion of the “cuteness” task, participants who were randomly allocated to the AF group received a unique password which enabled them to access the AF training material. This password restricted participation to those individuals who had been recruited, and ensured that the data from participants in the familiarisation group could be matched with their data from the AF training, which was delivered via Gorilla.sc. In order to maximise engagement, participants were informed that the amount of time they spent on each slide and set of slides would be monitored. Participants who appeared to have merely clicked through the slides with minimal engagement (less than 60 seconds of engagement; *n* = 2) were contacted via email and asked to read the slides more carefully. Participants were prevented from accessing more than one instalment per day via the use of a delay function within Gorilla.sc. Upon completion of the seventh and final instalment of AF training, participants received a final email containing a link and password to the “cuteness” task, and were encouraged to complete this final stage of the study as soon as possible (a similar email was sent to participants in the control group, a week after they had completed their baseline assessment). Participants in both groups received participation credit, with participants in the AF group also receiving a shopping voucher as reimbursement for the additional time spent during training. Finally, all participants were entered into a prize draw for shopping vouchers that was conducted upon completion of data collection.

### Data analysis

To assess the differences between groups we employed a linear mixed model (LMM) with fixed effects of group (Familiarised vs. Control), lip (CLP vs. TD), and time (Baseline vs. Post-test) and all interactions included as fixed effects.

Random effects of participant and image was included on the intercept. False Discovery Rate corrections [53] were used for multiple comparisons. Models estimated used restricted maximum likelihood and statistical values reported for individual model effects were obtained using the mixed() function of the “afex” package in R version 4.2.0 [54, 55], which obtained Type 3 tests by comparing models in which only the tested effect was excluded against the full model (including all effects) and using Satterthwaite denominator degrees of freedom.

## Results

Control group participants were found to have a mean(sd) age of 18.94(1.13) years with a mean age of 19.85(2.42) for AF group participants. At post-test the two groups did not show any difference in the average number of days spent completing the study (Control: M(sd) = 8.43(3.98); AF: M(sd) = 9.05(3.49)). Participants in the AF group spent on average(sd) 6.21(6.06) minutes on each daily training component, with an average(sd) total time of 43.47(23.05) minutes spent on the training as a whole. Given their potential effects on infant face perception and evaluation, the main effect of both participant and infant age and infant gender were controlled for in subsequent analyses. The effects of infant gaze and head off-centeredness were also accounted for.

For the tested LMM, the value of the conditional R Squared was 0.481 (48.09% of variance explained by the whole model) and the value of the marginal R Squared was 0.066 (6.58% of variance explained by fixed effects only).

**Fig 2:**
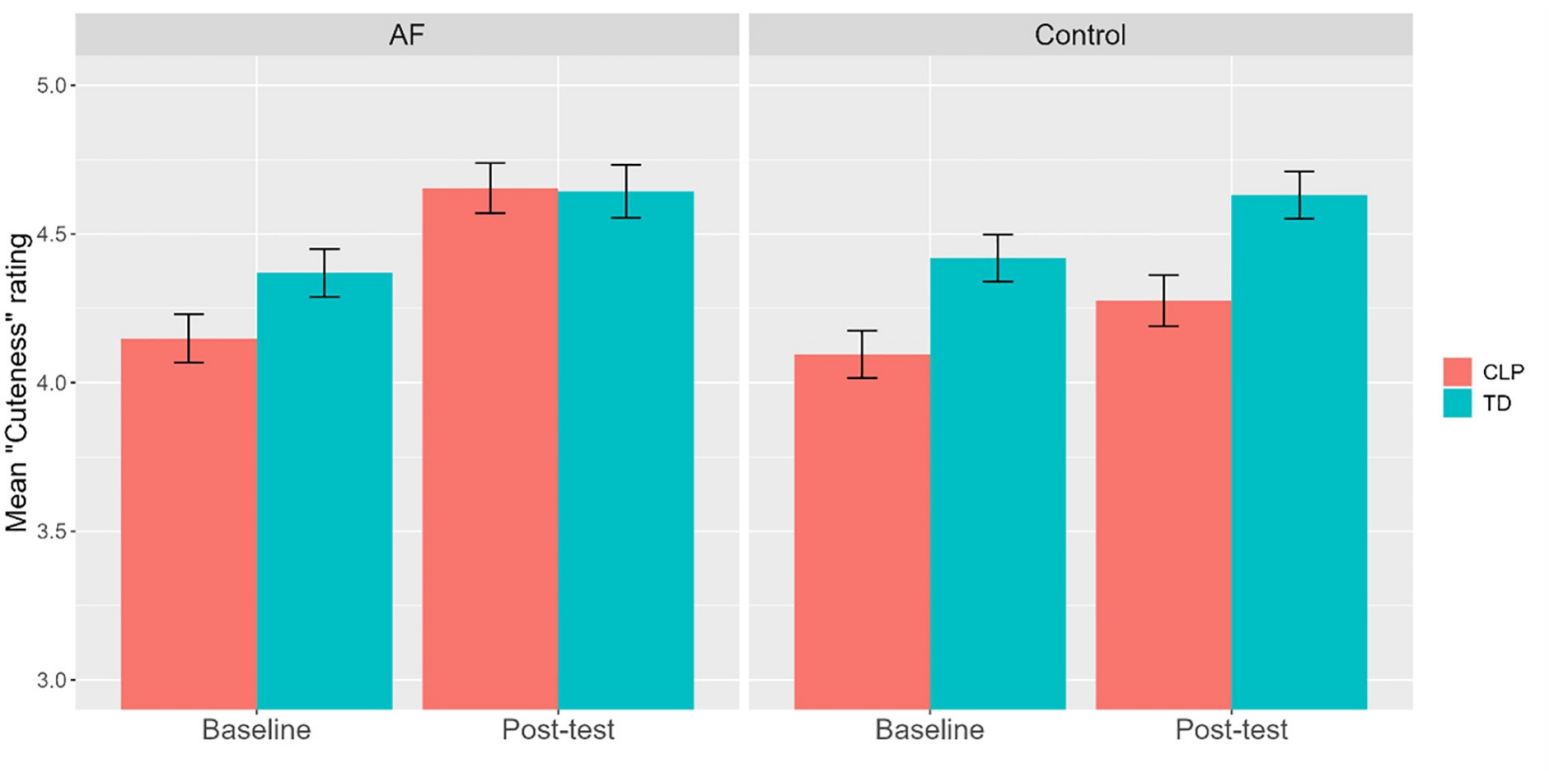
“Cuteness” rating means (with 95% confidence interval error bars), according to group, time and lip.

Directly addressing study hypotheses, a significant time*group*lip interaction (F(1, 8639.85) = 12.63, p < .001) was found (Fig 2) (findings on this and all other effects remained unchanged when excluding baseline data from participants with no post-test rating). Significantly higher ratings were provided at baseline for TD images compared to CLP images, by both control and familiarised participants (all p’s < .001). While this difference remained true (p < .001) at post-test for control participants, no difference was found between ratings of CLP and TD images for familiarised participants post-test. This was likely related to a significant increase from baseline to post-test in ratings of CLP images for familiarised participants (p < .001), who also showed a smaller, but significant increase in ratings of TD faces, between the two time points (p < .001). This is in contrast to control participants, who showed a slight, but significant increase in their ratings of TD images (p = .009), and no significant change between time points in their ratings of CLP images. In fact, in relation to group comparisons, control and familiarised participants showed no differences, at both time points, in their ratings of TD images, while ratings of CLP images were similar at baseline, but differed post-test, with familiarised participants showing higher ratings compared to control participants (p = .007).

Notably, while no effect of participant age on ratings was found, infant age had a significant effect (F(1, 43.01) = 5.71, p = .021), with ratings decreasing the older the infant pictured was. Infant gender also significantly affected ratings (F(1, 42.99) = 5.37, p = .026), which were found to be higher for female infants. Gaze (F(1, 43.00) = 17.29, p < .001) and head (F(1, 43.00) = 10.75, p = .002) direction were also found to have significant effects, but divergent directions: an increase in cuteness ratings was associated with increasing and decreasing off-centredness for gaze and head, respectively.

Given the variability reported above in the number of minutes spent by familiarised participants on the training, a secondary analysis was run to investigate the effect of training time on these participants’ ratings. This model included all covariates mentioned above, and the main and interactive effects of lip (TD vs. CLP) and training time (converted to 10-minute units). Training time did not significantly affect ratings for CLP (b = 0.10, SE = 0.06, p = .132) and TD (b = 0.06, SE = 0.06, p = .370) pictures, although the effect was stronger in the former (lip*training time: F(1, 1887.49) = 4.78, p = .029).

Given the presence of evidence for an increase in ratings for the control group as well, a further secondary exploratory analysis was conducted, to investigate the stability of ratings, from baseline to post-test, in the two groups, and for the two kinds of images (TD vs. CLP). For this purpose, a linear mixed model was run to predict post-test ratings, based on main and interactive effects of baseline scores, group and lip. A significant baseline score*group*lip interaction was found (F(1, 87.32) = 4.91, p = .029): while both groups, for both kinds of images, showed strong positive associations between baseline and post-test scores, only in the AF group was a significant difference between TD and CLP images found (b = -0.19, SE = 0.09, p = .037), with CLP images showing a decrease in the strength of this association.

Given its potential role in infant face perception, a final secondary analysis was conducted to investigate the effect of cleft severity on participant’s ratings. This was done by repeating the first reported model, but replacing the lip (TD vs. CLP) variable with one denoting cleft severity (TD vs. Low Severity vs. Medium Severity vs. High Severity) (once again, findings on model effects remained unchanged when excluding baseline data from participants with no post-test rating).

This second model confirmed all effects emerged from the first simpler model, including the same direction of effects. When comparing the two groups, at the different time points, no difference emerged between levels of severity, with one exception: high severity pictures received lower ratings than medium severity ones in the control group at baseline (p = .020), but this difference was not found post-test.

## Discussion

The present study aimed to explore the effects of familiarisation on how adult females perceive typical and atypical infant face configurations. To this end we explored whether female individuals who had been familiarised with infant CLP stimuli found CLP affected infants’ faces as attractive as those of TD infants, compared to naïve observers, who were hypothesised to find the latter more attractive than the former. Specifically, it was predicted that, before the familiarisation training, for both groups of participants, TD infants would be rated higher in “cuteness” than CLP infants, and that, only for those participants who had received the training, ratings for CLP infants would be higher after familiarisation, compared to before the training. Regarding the first hypothesis, as predicted, significantly higher ratings were provided for TD infants versus CLP infants at time 1 by both groups of participants, supporting findings from previous research that employed adult subjective ratings of infant CLP affected faces and found TD infants to be consistently rated higher than CLP infants on a variety of attractiveness indices (e.g., [26–28]).

With regards to the second hypothesis, as predicted, results showed a significant increase in “cuteness” ratings after familiarisation, only for AF participants, with this effect not being observed in participants who had not undergone AF training.

Investigations into the Kindchenschema have typically involved manipulation of infant facial features, with comparisons being made, for example, between infants with low and high baby schema (e.g. [16, 20, 56]). Infant-related differences, such as gender [8], and degree of “cuteness” (increased “cuteness” intended as degree of correspondence with baby schema) [16] have frequently been found to influence adult judgments of attractiveness, with female infants, and “cuter” ones receiving the highest attractiveness ratings. Few studies investigating Kindchenschema have explored the effects of participant characteristics. For example, age and sex of the adult observer have previously been explored as predictors of subjective infant attractiveness ratings [11, 29, 57], with younger females typically providing higher ones than older females and males. Even fewer studies have explored the manipulation of participant experiences, with an example being a study conducted by Parsons et al. [33], who influenced participant experience by experimentally manipulating infant temperament. The researchers found attractiveness ratings could be increased or attenuated in participants through pairing emotionally neutral infant images with positive or negative infant facial expressions and vocalisations during a training phase. The present study adds to this still relatively unexplored area of study, showing how adult perceptions and evaluations of infant features are not uniquely tied to the specific infant face and its degree of adherence to the “baby schema”, but remain susceptible to change as a consequence of experience.

Research has found that, like adults [58], attractive infants fare better than less attractive infants in a variety of social contexts. For example, attractive children are judged as being healthier and happier [59] which is likely to have an effect on how motivated adults are to interact with them [60]. In addition, more attractive infants have been found to receive more affectionate behaviour from their mothers [61] and tend to receive higher academic performance ratings from adults in educational environments [62]. Clearly then, if infant attractiveness can be influenced by factors other than the infants’ own appearance, further understanding of the wider promoters of infant attractiveness have importance for infant development and experience. Moreover, given the wide range of possible neonatal facial malformations, future studies may also aim to investigate whether the effects reported in the present study obtain in the context of malformations other than CLP. If the present findings, showing familiarity to attenuate the effects of CLP on adult evaluations of infant attractiveness, were replicated in relation to other conditions, this may prove of particular practical and clinical significance in providing support to new and expectant parents of infants with a diagnosed malformation. It may also have clinical public health implications and inform policies for the wider promotion of material aimed at familiarising the general public with malformations such as CLP, with the ultimate goal of improving their public perception.

Focusing on the specific alteration to the Kindchenschema brought about by the presence of a CLP, and what effects this may have on the adults caring for these infants, the present findings could prove especially informative. Previous studies (see, for example, [63]) have reported the detrimental effects of infant CLP on adult evaluations and reactions, noting the need to investigate the role of these in the context of the often-reported difficulties in early interactions between parents and their CLP-affected infant [64, 65].

Far from negating the need for these studies on parent reactions, the present findings would seem to point to the fact that a mother’s likely familiarity with their infant’s CLP may offer them some degree of protection from the negative effects of this Kindchenschema alteration, and that the early interactive difficulties may be related to the presence of other factors (e.g., parental appraisal of the cleft, see [4]). Notably, in the present study, this protective effect proved to be independent of the level of cleft severity, which previous studies found to be an additional determinant of adult reactions, with higher levels of severity tied to more negative evaluations of infant faces [28]. Admittedly, however, the number of images within each severity classification were not balanced, warranting some caution in interpreting related findings, and highlighting the need for further dedicated investigations.

The finding that familiarity attenuated responses to Kindchenschema alterations may appear especially striking, considering previous findings evidencing stronger, rather than attenuated, responses to images of infants in mothers compared to non-mothers, when the latter may be presumed to be more familiar with infant stimuli than the former [41, 42, 66]. This may be related to the fact that these previous studies compared reactions to infant and adult faces, and not clear disruptions to the typical configuration of infant facial features: if, in caregivers, stronger reactions to infant stimuli, compared to adult ones, may prove advantageous for offspring survival, a mechanism, such as the one tied to familiarity, which maintains attraction to infant stimuli in the face of alterations to the Kindchenschema, may prove similarly advantageous. Future studies may focus on disentangling the effects of familiarity and of the parental bond, to further investigate these mechanisms.

The recommendations made by Parsons and colleagues [63], to study these effects in parents are therefore reiterated here. In particular, recent findings [26] have shown how the presence of infant CLP is associated with reduced amplitude in the P1 and N170 event related potential components, tied to the processing of faces, and, crucially, how this reduced amplitude explains the lower attractiveness ratings for infants with a cleft lip compared to healthy infants. Following the present findings, future studies may wish to utilise electroencephalography to investigate whether, in the context of high familiarity with CLP, these relationships and explanatory pathways between event related potentials and attractiveness ratings still obtain, given the increased level of cognitive appraisal involved in the latter.

Rayson and colleagues [28] also reported that, for both TD and CLP infant faces, attractiveness ratings negatively correlated with the time participants spent looking at the mouth area vs. the eye area of the face. This is especially notable, given that mothers of infants born with CLP were found to spend significantly less time than mothers of TD infants gazing at their infant’s mouth [34]. While this difference in findings, as noted at the start of the present paper, is probably also tied to macroscopic differences between these two groups (e.g., parental appraisal of the cleft, parental link to the infant, general parenthood), which should be fully researched, future investigations, through methods relying on eye-tracking technologies, could also test whether the aforementioned potential protective effect of familiarity (possibly the most basic factor shared by the two groups) may be explained by specific alterations in gaze patterns, which see attention diverted from the cleft-affected areas of the infant face. Such a finding would prove of great clinical importance, given that reduced visual attention to the mouth area in the context of CLP has been found to reduce the rate of maternal mirroring responses, key promoters of infant social development [4].

Of note, we also found slight but significant increases in ratings of TD infants between the two time points for both groups of participants. Whilst not explicitly predicted, this finding is in keeping with the broader literature investigating the mere exposure effect (e.g., [67, 68]) which suggests that individuals show enhanced attitude for items that they have experience or prior familiarity with [69]. In the case of this study this enhanced attitude was reflected in increased “cuteness” ratings for typical infants upon their second presentation, and possibly account for the small and non-significant increase in ratings of CLP infants for control participants.

Admittedly, this finding suggests some level of familiarity occurred as a result of exposure to the baseline task, perhaps for both groups of participants. As control participants were not subjected to an equivalent task, we are unable to determine whether the increases in ratings from pre to post-test for CLP infants are related to repeated testing. A future study may wish to engage control participants in a similar task with a parallel arm (e.g., familiarisation with visual and informational content related to typical infants) to better elucidate these findings.

In an interesting secondary study finding, age of target infant was found to be negatively related to “cuteness” ratings, with younger infants receiving higher ratings. This finding is broadly in keeping with research conducted by Glocker et al. [16] and Parsons et al. [63] who reported higher attractiveness ratings for infants higher on the baby schema spectrum. Whilst we did not manipulate the facial features of the infant images as in the aforementioned studies, age has been found to be related to Kindchenschema in past research [11] that reported “cuter” infants to be judged as younger than less “cute” infants. Given the likely negative relationship between an individual’s age and their vulnerability and need for care (i.e., the younger the infant is, the more in need of caregiving behaviour it is), the greater attractiveness perceived in younger infants may be an especially advantageous Kindchenschema-related effect, in terms of species survival.

Both gaze and head direction were also found to be significantly associated with “cuteness” ratings, with ratings being positively associated with increasing off-centredness of eye gaze, and decreasing off-centredness of head direction. Our result at least partly contrasts with the findings of both Mason et al. [14] and Ewing et al. [13] who reported increased likeability and attractiveness ratings by adult observers of adult target faces where these were looking nearer to centre. While no specific hypothesis was suggested, in the current study, in relation to these variables, these findings would seem to point to the opportunity for future studies of exploring the role of head and gaze direction in the perception of infant vs. adult faces.

Furthermore, the present study focused on a specific population, namely female individuals who were not parents. This was done because previous research on gender differences in response to infant faces has produced mixed findings (e.g., [20, 21, 57]), and due to potential confounds associated with responses to infants by parents compared to non-parents [40, 70]. Nonetheless, future research may further explore current findings through the lens of sex differences and accounting for a more fine-grained measure of participant general experience with young children in general.

The present study had a number of strengths. The AF training materials were specifically designed for the present study, and their sole purpose was to produce a level of familiarity with infant CLP in a previously naïve group of female participants. Based on our analyses, the AF training was found to be an effective tool for this purpose, and might be used in future studies that aim to explore the role of prior familiarity with infant CLP on adult-infant interaction.

Previous research on attractiveness ratings of adult faces has found an effect of facial off-centredness [13], and to account for these variations between stimuli, and representing a further study strength, we controlled for both eye and head direction of each infant face in the statistical analyses, as per Rayson et al. [28].

There were, however, a number of limitations. While the AF training was found to successfully affect participants’ evaluations of infant faces, it was not designed to enable investigation of which of its elements (i.e., visual or textual) exerted this effect, or how much exposure was necessary for the observed familiarising effect to manifest itself. Future research may focus on exploring these aspects in greater detail. A better understanding of these factors may also inform the design and use of educational material for parents who are expecting or who have had an infant born with a CLP.

In addition, participants were generally left free to access the training materials at their own convenience, and, as a result, a small number of participants exceeded the prescribed seven days of training duration. Future studies adopting this training approach may opt for stricter forms of control of participant engagement. Nonetheless, given that exposure time to the AF training materials was variable as a result of this participant-initiated engagement with the training phase, we explored the possibility of the effect of training time on “cuteness” ratings in the AF group. Notwithstanding a significant interaction, pointing to a stronger effect in relation to CLP compared to TD images, probing this interaction evidenced no significant effect of training time on ratings of either TD or CLP images.

A further limitation was our employing only one outcome variable, namely, responses to the “cuteness” rating question. Although previous studies have taken this same approach (e.g., [8, 20, 56]), the inclusion of a single outcome variable may undermine confidence in findings and conclusions. Related research has used other, similar outcomes in addition to “cuteness” rating, such as infants’ perceived need for care and protection [31], and appraisal of infant mood [11]. Inclusion of these measures alongside “cuteness” ratings in future research might therefore strengthen findings.

To summarise, the present study explored how typical and atypical infant face configurations are perceived by adult female individuals, following a week-long training phase, where participants were familiarised with CLP, the specific atypical facial configuration tested. While at baseline, infants affected by CLP were rated significantly lower than TD infants on indices of “cuteness” by both groups of participants, participants who underwent the training demonstrated increased acceptance of infant CLP, demonstrated by increased attractiveness ratings, at the post-test assessment. While most previous studies on adult ratings of infant faces have looked at the effects of modifying facial features of the input stimuli, the present study is one of a small number showing how adult evaluations and reactions to infant faces can be modified by manipulating the participants’ experience. More specifically, study findings point to familiarity as a protective factor against the negative effects of typical face configuration alterations on adult perception of infant faces.
